# Supplementary material for: Splice-Junction-Based Mapping of Alternative Isoforms in the Human Proteome
Source: Cell Rep. Author manuscript; Available in PMC 2020 Jan 15. (PMC6961840; doi:10.1016/j.celrep.2019.11.026)
Supplement: 2 [file NIHMS1546469-supplement-2.zip › DF1/PXD010154/Testis/Testis_42_ZNF212_GGLALEPGRPNGLVSPSR.pdf]

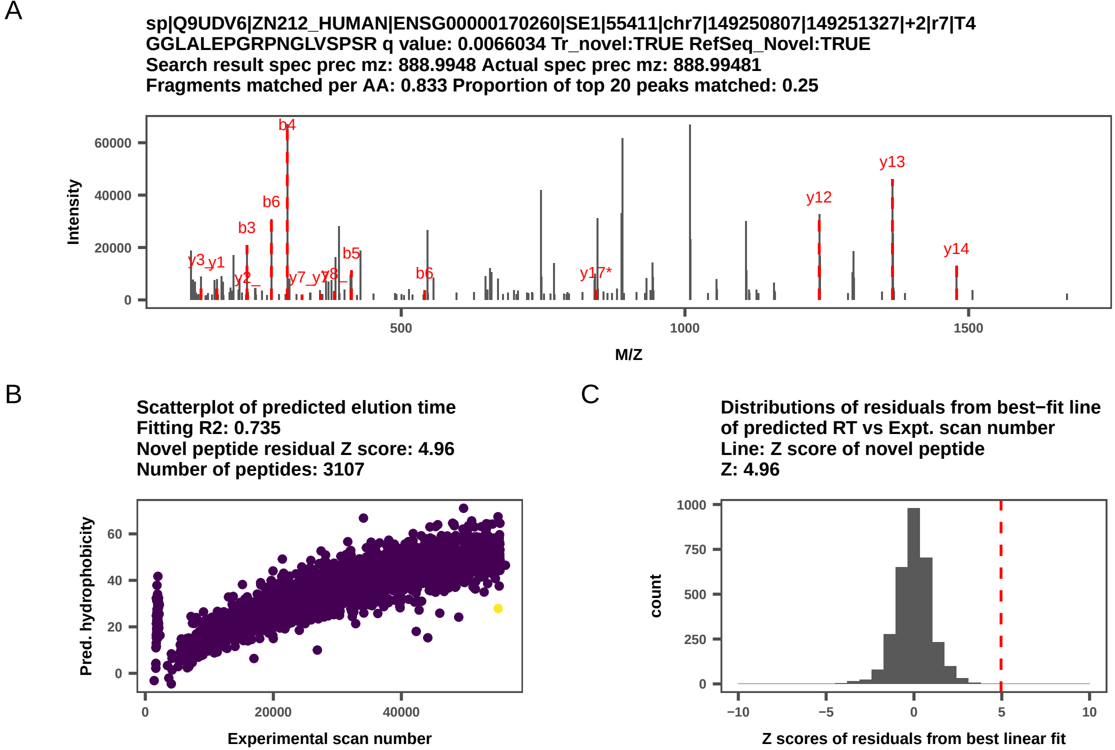
A
sp|Q9UDV6|ZN212_HUMAN|ENSG00000170260|SE1|55411|chr7|149250807|149251327|+2|r7|T4
GGLALEPGRPNGLVSPSR q value: 0.0066034 Tr_novel:TRUE RefSeq_Novel:TRUE
Search result spec prec mz: 888.9948 Actual spec prec mz: 888.99481
Fragments matched per AA: 0.833 Proportion of top 20 peaks matched: 0.25
Intensity
60000
40000
20000
0
b4
b6
b3
y3
y1
y2
y7
b5
b6
y17*
y12
y13
y14
500
1000
1500
M/Z
B
Scatterplot of predicted elution time
Fitting R2: 0.735
Novel peptide residual Z score: 4.96
Number of peptides: 3107
Pred. hydrophobicity
60
40
20
0
20000
40000
Experimental scan number
C
Distributions of residuals from best−fit line
of predicted RT vs Expt. scan number
Line: Z score of novel peptide
Z: 4.96
count
1000
750
500
250
0
−10
−5
0
5
10
Z scores of residuals from best linear fit
